# Measuring depression severity based on facial expression and body movement using deep convolutional neural network

**DOI:** 10.3389/fpsyt.2022.1017064

**Published:** 2022-12-21

**Authors:** Dongdong Liu, Bowen Liu, Tao Lin, Guangya Liu, Guoyu Yang, Dezhen Qi, Ye Qiu, Yuer Lu, Qinmei Yuan, Stella C. Shuai, Xiang Li, Ou Liu, Xiangdong Tang, Jianwei Shuai, Yuping Cao, Hai Lin

**Affiliations:** ^1^Department of Physics, Fujian Provincial Key Laboratory for Soft Functional Materials Research, Xiamen University, Xiamen, China; ^2^Department of Psychiatry, National Clinical Research Center for Mental Disorders, The Second Xiangya Hospital of Central South University, Changsha, China; ^3^Department of Psychiatry, Baoan Mental Health Center, Shenzhen Baoan Center for Chronic Disease Control, Shenzhen, China; ^4^Oujiang Laboratory (Zhejiang Lab for Regenerative Medicine, Vision and Brain Health), Wenzhou Key Laboratory of Biophysics, Wenzhou Institute, University of Chinese Academy of Sciences, Wenzhou, Zhejiang, China; ^5^Integrated Chinese and Western Therapy of Depression Ward, Hunan Brain Hospital, Changsha, China; ^6^Department of Biological Sciences, Northwestern University, Evanston, IL, United States; ^7^Sleep Medicine Center, Mental Health Center, Department of Respiratory and Critical Care Medicine, State Key Laboratory of Biotherapy, West China Hospital, Sichuan University, Chengdu, China; ^8^State Key Laboratory of Cellular Stress Biology, Innovation Center for Cell Signaling Network, National Institute for Data Science in Health and Medicine, Xiamen University, Xiamen, China

**Keywords:** smart medical, depression, behavioral entropy, deep learning, artificial intelligence

## Abstract

**Introduction:**

Real-time evaluations of the severity of depressive symptoms are of great significance for the diagnosis and treatment of patients with major depressive disorder (MDD). In clinical practice, the evaluation approaches are mainly based on psychological scales and doctor-patient interviews, which are time-consuming and labor-intensive. Also, the accuracy of results mainly depends on the subjective judgment of the clinician. With the development of artificial intelligence (AI) technology, more and more machine learning methods are used to diagnose depression by appearance characteristics. Most of the previous research focused on the study of single-modal data; however, in recent years, many studies have shown that multi-modal data has better prediction performance than single-modal data. This study aimed to develop a measurement of depression severity from expression and action features and to assess its validity among the patients with MDD.

**Methods:**

We proposed a multi-modal deep convolutional neural network (CNN) to evaluate the severity of depressive symptoms in real-time, which was based on the detection of patients’ facial expression and body movement from videos captured by ordinary cameras. We established behavioral depression degree (BDD) metrics, which combines expression entropy and action entropy to measure the depression severity of MDD patients.

**Results:**

We found that the information extracted from different modes, when integrated in appropriate proportions, can significantly improve the accuracy of the evaluation, which has not been reported in previous studies. This method presented an over 74% Pearson similarity between BDD and self-rating depression scale (SDS), self-rating anxiety scale (SAS), and Hamilton depression scale (HAMD). In addition, we tracked and evaluated the changes of BDD in patients at different stages of a course of treatment and the results obtained were in agreement with the evaluation from the scales.

**Discussion:**

The BDD can effectively measure the current state of patients’ depression and its changing trend according to the patient’s expression and action features. Our model may provide an automatic auxiliary tool for the diagnosis and treatment of MDD.

## Highlights

- Multi-modal network is more effective to detect depression.
- A novel metrics is proposed to evaluate the depression severity.
- Patients showed more sad expression and a smaller range of body movements.

## Introduction

With a high prevalence, high recurrence rate, high disability rate, and high fatality rate, major depressive disorder (MDD) is becoming a leading issue in the global burden of disease (1). According to statistics from the World Health Organization (WHO), there are approximately 350 million people suffering from depression worldwide, and about one million people who commit suicide as a result of depression every year (2). According to the Global Burden of Disease Survey, depression has the largest weight in the burden of mental illness in terms of disability-adjusted life years, accounting for about 40.5% (3, 4). The latest epidemiological survey conducted in 31 provinces in China showed that the lifetime prevalence of depression is 6.8% (5). Depression severely affects quality of life (6) and brings heavy mental and economic burdens to the family structures and society as a whole.

So far, the cause of depression is ambiguous. It is only confirmed that depression is associated with biological, psychological, and social environmental factors. The clinical diagnosis of depression is mainly based on a doctor’s interview combined with some psychological rating scales to make a comprehensive assessment. At present, however, there is an extreme shortage of mental health professionals in developing countries, such as China, resulting in poor access to professional medical services for depression, a lack of timely assessment of symptoms, and greatly increased chances of depression relapse.

The rise of artificial intelligence (AI) based on big data analysis brings promising advancements to doctors and patients. With the fourth industrial revolution initiated in the 21st century, AI has increasingly been utilized in the research of mental diseases (7). Machine learning models can provide an accurate prediction of the onset of depressive disorder for patients (8–14). The diagnostic model includes age measurement, a simplified mental state examination score, and structural imaging. The treatment response model includes measures of structural and functional connectivity.

Deep representation features, such as body movements, gestures, eye movement, and periodic muscle movement, can be used for the biological analysis of depression. The analysis includes the recognition, judgment, tracking, and understanding of human behaviors (15–17). An individual can produce specific expressions through certain forms of muscle contraction, and these expressions can be used as a carrier of emotions, intentions, desires, and other information (18, 19). The deep convolutional neural network (CNN) uses these biological signals to identify depression and has achieved promising results (20–29). In this way, an AI-based solution to assess symptom severity may be utilized for the assessment and auxiliary diagnosis of patients with depression. By making individual and objective predictions on mental disorders, we can improve the accuracy of patient diagnosis and treatment decisions. The application of AI over the whole course of depression will profoundly affect the evaluation, prediction, and treatment of depression (30).

Most of the previous AI models focus on single-modal information of a certain feature of the patient. In recent years, some studies have shown that multi-modal information has a better prediction performance than single-modal data (20, 31). In this paper, we proposed a multi-modal deep convolutional neural network (CNN) model based on facial expressions and body movements to evaluate the severity of depression. The model included two modules: an expression recognition module to predict the probability of 7 facial expressions of patients, using expression entropy to represent the complexity of facial expressions, and a movement recognition module to locate the position of 18 body joints of the patient, using action entropy to represent the complexity of the body movement. According to the expression and movement features output by the model, a behavioral depression degree (BDD) was derived to quantify the severity of depression, which was used as the detection result of our model. Our results demonstrated an over 74% Pearson similarity between BDD and self-rating depression scale (SDS), self-rating anxiety scale (SAS), and Hamilton depression scale (HAMD), indicating that the BDD can effectively measure the current state of patients’ depression and its changing trend according to the patient’s expression and action features.

## Materials and methods

Prospective and qualitative research methods were used in this study. In order to obtain the continuous change process of the patient’s facial expressions and movements, the patients with MDD who received any kinds of psychotherapy and/or pharmacotherapy were selected, and the entire process was recorded with 40–60 min of video recording in the symptoms assessment every week. Two patients were randomly selected for receiving systematic cognitive behavioral treatment (CBT) for seven sessions from the subject pool, and video data was collected during the whole process of psychotherapeutic interviews. Each psychological assessment was conducted in a fixed room, and psychotherapy was conducted in a quiet treatment room with soft lighting, to ensure undisturbed talks. During the psychological assessment, the camera targeted at the sitting subject’s face and whole body. During the psychotherapy, the therapist and the subject seated at an angle of 90–120°, and a camera lens targeted at the subject’s face and whole body. We used the Shadow Giant portable 4K camera with 2560*1440/30fps resolution for recording, and all videos were saved as “mov” or “mp4” format in hard disk. The video data was processed by a computer server with Intel Xeon 3.20GHz CPU, 500G SSD, 2T HHD, 32GB RAM, and two GTX 1080Ti GPU.

We used behavioral entropy (BE) to describe the degree of discreteness in patients’ facial expression and action features. We then established the behavioral depression degree (BDD) based on BE to represent the degree of depression we assessed.

### Subjects

Patients with MDD who would receive inpatient and outpatient treatment for at least 2 consecutive weeks were recruited from the Second Xiangya Hospital of Central South University and Hunan Brain Hospital of Hunan Province from August 2020 to January 2021. Each patient was diagnosed by a senior and an attending psychiatrist at the same time. The inclusion criteria were as follows: (i) all participants aged between 16 and 50 years; (ii) all participants met DSM-IV criteria for MDD; (iii) their Hamilton depression rating for depression 24-item total score was not less than 18; and (iv) all participants were free of any antidepressant and antipsychotic at least 1 month before trial. The exclusion criteria included (i) a history of dysthymia, mania, hypomania, bipolar disorder, or depression secondary to a known substance or general medical condition; (ii) a drug allergy or clinically significant laboratory abnormality; (iii) active suicidal ideation, comorbid mental disorders, catatonic features, or severe psychomotor retardation that made interviewing difficult; (iv) active substance abuse; (v) a history of brain injury or other severe medical comorbidity, such as stroke, diabetes, or cardiovascular disease; (vi) current medication that might influence mood or the central nervous system; and (vii) pregnancy or breastfeeding.

This study was approved by the Medical Ethics Committee of Second Xiangya Hospital of Central South University in China. Each patient was given assessment once a week for at least two times, while a video recording was kept for each assessment. All patients provided written informed consent prior to their participation in the study.

### Measurements

All patients were assessed *via* the following questionnaires at baseline and before hospital discharge, or at weekly symptom assessment. The two selected patients were assessed after each psychotherapy session.

#### Self-rating depression scale (SDS)

The SDS consists of 20 items which can be used to assess the severity of depression symptoms and the changes in patients’ symptoms during treatment. SDS uses a four-level scoring method of 1–4 points (none or occasionally, sometimes, often, always). Those with a score below 50 have no depression while 50–59 represents mild depression, 60–69 represents moderate to severe depression, and 70 or higher represents severe depression.

#### Self-rating anxiety scale (SAS)

The SAS is similar to the SDS and consists of 20 items on a four-point scale from 1 to 4 (none or occasionally, sometimes, often, always). A score below 50 indicates no anxiety, 50–59 indicates mild anxiety, 60–69 indicates moderate to severe anxiety, and over 70 indicates severe anxiety.

#### Hamilton depression scale (HAMD)

Hamilton depression scale uses a five-point scale of 0–4 (none, mild, moderate, severe, very severe). The HAMD total score can better reflect the severity of the disease. The more severe the symptoms are, the higher the total score is, as a score more than 35 points denotes severe depression, 18–35 points denotes mild or moderate depression, and less than 8 points denotes no depression symptoms.

### Model analysis

A multi-modal deep CNN was used to identify facial expressions and body motion range of the patients to obtain their expression and action features. Then the difference between the predicted results of the model (BDD) and the total scores of SDS, SAS, and HAMD were used to judge the prediction accuracy of the model.

**FIGURE 1:**
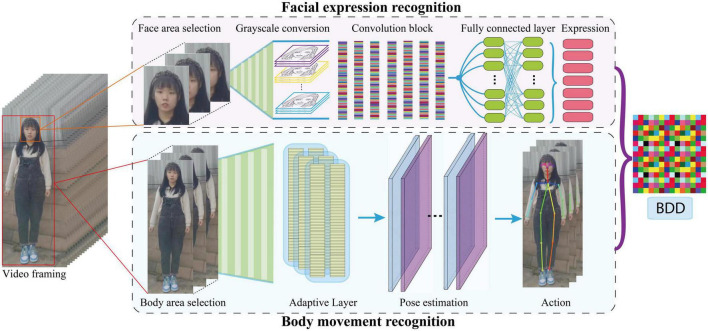
Overview of the proposed model.

Figure 1 shows the overview of the proposed model framework. The video data were preprocessed and fed into the expression and body recognition modules. The expression module includes face detection and facial expression recognition. We used open-source Python package DBFace^1^ for face detection. Then we constructed a deep convolution neural network (Table 1) and trained a facial expression recognition module using Facial Expression Recognition 2013 Dataset (Fer2013),^2^ which contains about 30,000 facial images, with each image labeled as one of the seven universal facial expressions (32) (angry, disgust, fear, happy, sad, surprise, neutral). In the action module we use the open-source Python package tf-pose-estimation,^3^ which includes human body region recognition, adaptation layer, and pose estimation based on TensorFlow (33). The position of the 18 nodes of the human body is output by this module. Finally, our model outputs the BDD which represents the patient’s behavioral depression degree.

**TABLE 1:** The convolutional neural network for facial expression recognition.

Convolution block	Output shape	Layer
conv2d	48 × 48 × 64	batch_normalization
		Activation
conv2d_1	24 × 24 × 64	batch_normalization_1
		activation_1
		max_pooling2d
conv2d_2	24 × 24 × 128	batch_normalization_2
		activation_2
conv2d_3	12 × 12 × 128	batch_normalization_3
		activation_3
		max_pooling2d_1
conv2d_4	12 × 12 × 256	batch_normalization_4
		activation_4
conv2d_5	6 × 6 × 256	batch_normalization_5
		activation_5
		max_pooling2d_2
conv2d_6	6 × 6 × 512	batch_normalization_6
		activation_6
conv2d_7	3 × 3 × 512	batch_normalization_7
		activation_7
		max_pooling2d_3
	1 × 1 × 7	dropout, 2-d fc, softmax

#### Facial expression recognition

Facial expression recognition can be used to screen for mental illness, assess the effectiveness of medication, and assess the progression and severity of mental illness (34, 35). In our deep CNN, we carried out operations such as convolution, pooling, and activation, on the input image of 48*48 pixels, and finally added a full connection layer to output the seven facial expressions of the patient. At the same time, to prevent the model from overfitting, we added a dropout layer for discarding. The network architecture is shown in Table 1. We trained our CNN network using Fer2013 dataset and achieve 70.71% accuracy in the task of facial expression recognition, which exceeds the human accuracy of 65% (36). After using DBFace to detect the patient’s face from the psychotherapeutic interviews video, we then used our trained model to estimate the patient’s expression and obtained the probability of seven expressions of the patient’s face in each frame of the video.

Finally, we introduced the expression entropy through the following:


(1) $$\begin{array}{c} H\;\left(X\right)=-\sum_{1}^{7}p\;\left(x_{i}\right)\;l\,o\,g\,p\;\left(x_{i}\right) \end{array}$$

H(X) is the expression entropy, xi is the i-th expression, and p(xi) is the probability of the i-th expression calculated by the facial expression recognition model. The expression entropy reflects the degree of discreteness of the patient’s expression. A high value indicates that the patient’s expression is rich and lively. On the contrary, a low value indicates that the patient’s expression is relatively simple and the degree of depression is more serious (16, 37).

#### Body movement recognition

In the field of computer vision, human pose estimation refers to the process of determining positioning information, such as the position and direction of different human parts in pictures, through image analysis (38). Human pose estimation is the basis of human behavior recognition (39). In this module we applied tf-pose-estimation to locate the position of 18 joints of the target person in the video. Then, according to the position changes of the joint points between two pictures, we use the two coordinate positions of the same joint point in the pictures to calculate the motion amplitude using the equation:


(2) $$\begin{array}{c} D=\sqrt{{\left(x_{1}-x_{0}\right)}^{2}+{\left(y_{1}-y_{0}\right)}^{2}} \end{array}$$

D represents the motion amplitude of the joint point while (x_0_, y_0_) and (x_1_, y_1_) represent the position of the same joint point in the two pictures.

Finally, we introduce the action entropy:


(3) $$\begin{array}{c} G\;\left(Y\right)=-\sum_{j=0}^{17}\frac{D\,\left(y_{j}\right)}{D_{z}}\,l\,o\,g\,\frac{D\,\left(y_{j}\right)}{D_{z}} \end{array}$$

$D_{z}=\sum_{j=0}^{17}D\,\left(y_{j}\right)$, G(Y) is the action entropy, y_*j*_ represents the j-th joint point, and D(y_*j*_) is the motion amplitude of the j-th joint point calculated by limb recognition module.

#### Behavioral depression degree (BDD)

We linearly superimpose the entropy of expression and action into BE:


(4) $$\begin{array}{c} F\;\left(X,\;Y\right)=\lambda \,H\;\left(X\right)+\left(1-\lambda \right)\;G\;\left(Y\right) \end{array}$$

F(X, Y) stands for the BE, H(X) for expression entropy, G(Y) for action entropy, X for expression features output by the model, Y for action features output by the model, and λ for a fitting parameter. Then we scan λ from 0 to 1, which adjusts the proportion of expression entropy and action entropy in BDD to find the best parameter λ that allows BDD to best match with SDS, SAS, and HAMD. The BE describes the disorder degree of patients’ expressions and movements. Here, we assume that the larger the expression entropy is, the livelier the patients’ expressions are (16, 37), and the larger the action entropy is, the larger range of patients’ movements is, indicating a less depression condition (40).

We then introduce behavioral depression degree (BDD) to represent the patient’s depression degree predicted by the model:


(5) $$\begin{array}{c} B\;\left(X,Y\right)=1-F\;\left(X,Y\right) \end{array}$$

B(X, Y) represents the patient’s BDD predicted by the model and F(X, Y) represents the BE.

## Results

### General condition

A total of 164 patients with MDD fit for inclusion criteria were recruited in this study, all of them aged 17–35, with 12–18 years of education. The data of two patients (patient 1 and patient 2) who were randomly selected for the 7-session systemic psychotherapy were used for the BDD score analysis.

#### Selected patient 1

Patient 1 is a 20-year-old unmarried female college student. She complained of too much work, stress, and depression for at least half a year. In July 2020, she went to Hunan Brain Hospital for treatment and was diagnosed with “depression,” and treated with “agomelatin” 50 mg/Qn. Nearly 1 month later, on the recommendation of the attending doctor, she began to receive psychological treatment. When the patient came for psychotherapy, she was in her third year of college study. She needed to balance her learning tasks, internship, and preparation for postgraduate entrance exams at the same time. She felt great pressure and experienced stressful life events such as breaking up with her boyfriend and her grandmother’s death.

#### Selected patient 2

Patient 2 is an 18-year-old female who is unmarried and currently in senior year in high school. She complained of decreased sleep quality half a year ago, difficulty in falling asleep, early awakening, wanting to cry for no reason, and being easily agitated with suicide ideation. In October 2020, she went to Hunan Brain Hospital accompanied by her father and was diagnosed with “major depressive disorder.” Her father was informed about the risk of self-injury and suicide after being unwilling to accept hospitalization, and the patient was treated with “Fluoxetine Hydrochloride Capsule” 20 mg/Qd. The next day, under the advice of the attending doctor, the patient began to receive psychotherapy.

The type and dose of drugs for the two patients remained the same throughout the study period.

### The total scores of SDS, SAS, and HAMD of the patients

#### Selected patients

**FIGURE 2:**
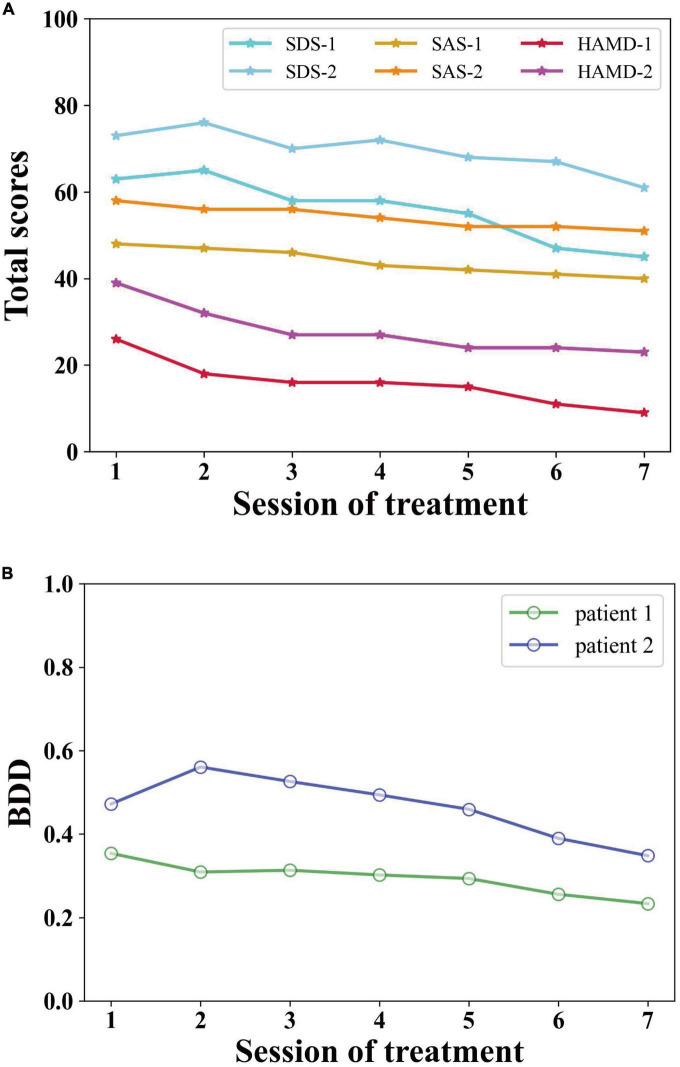
The total scores of **(A)** SDS, SAS, HAMD, and **(B)** BDD of the two selected patients.

As shown in Figure 2A, the SDS total score of patient 1 dropped from 63 at the end of the first treatment to 45 at the end of the seventh treatment, below the clinical threshold (>50). The SDS index decreased by 28.6%. The SAS, as a self-rating scale, has a strong correlation with the SDS. The SAS total score of patient 1 dropped from 48 at the end of the first treatment to 40 at the end of the seventh treatment, below the clinical threshold (>50). The SAS index decreased by 16.7%. The HAMD total score of patient 1 dropped from 26 at the end of the first treatment to 9 at the end of the seventh treatment, a score of which belongs to mild depression. The HAMD index of patient 1 decreased by 65.4%.

The SDS total score of patient 2 decreased from 73 at baseline to 61 at the end of the seventh treatment, with a slight decrease of 16.4% compared with baseline, but still higher than the clinical threshold (>50). The SAS total score of patient 2 decreased from 58 at baseline to 51 at the end of the seventh treatment, with a slight decrease of 12.1% compared with baseline, but still higher than the clinical threshold (>50). The HAMD total score of patient 2 decreased from 39 at baseline to 23 at the end of the seventh treatment which belongs to moderate depression. Compared with baseline, the HAMD index of patient 2 decreased by 41.0%.

### Expression features of the two selected patients

**FIGURE 3:**
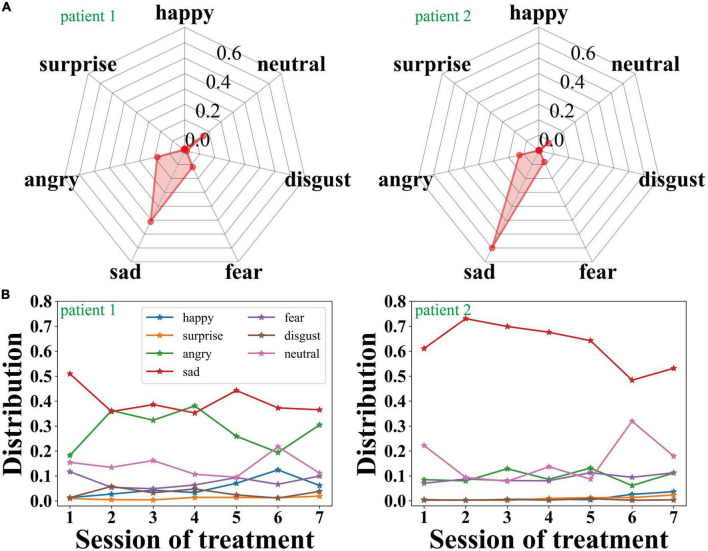
**(A)** The expression feature distributions of the two selected patients during the second session of treatment, and **(B)** the change of the expression features of the two selected patients with the sessions of treatment.

Figure 3A shows the expression distribution of patient 1 and patient 2 during the second session of treatment. The larger the upper shadow area is, the better the emotional state is, while the lower shadow represents bad emotions. We can see that the proportion of sad and angry expressions is significantly higher than other expressions.

As shown in Figure 3B, throughout the entire 7 sessions of treatment, the proportion of sad and angry expressions of patient 1 is relatively high but gradually decreases, and the emotional state of the patient has been significantly improved as the treatment progressed. Meanwhile, patient 2’s sad expression accounts for a larger proportion in the entire psychotherapy. Throughout the process of the treatments, the proportion of sadness gradually decreases, which reveals that the emotional state of patient 2 has been improved to a certain extent.

### Action features of the two selected patients

**FIGURE 4:**
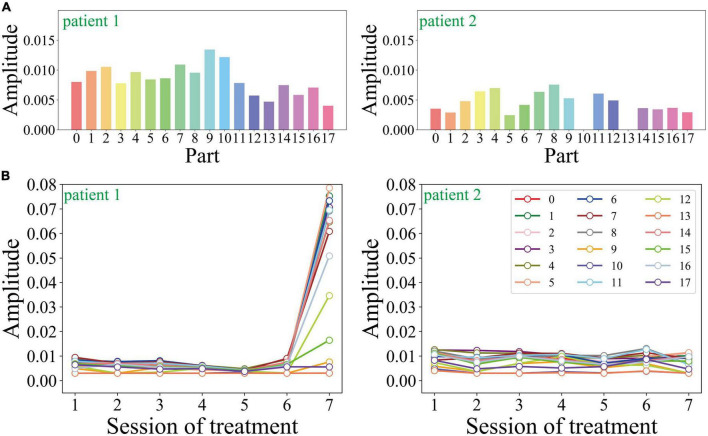
**(A)** The action feature distributions of the two selected patients during the second session of treatment, and **(B)** the change of the action feature with the sessions of treatment.

As shown in Figure 4A, we compare the amplitude of the two patients’ limbs in the second session during the treatment process. 0–17 represents nose, neck, right shoulder, right elbow, right wrist, left shoulder, left elbow, left wrist, right hip, right knee, right ankle, left hip, left knee, left ankle, right eye, left eye, right ear, and left ear. The average amplitude of each joint in patient 1 is 0.0084, and that of patient 2 is 0.0042. Additionally, the joint motion range of patient 2 is significantly lower than that of patient 1. As for the empty joints in the picture (such as patient 2’s right ankle and left ankle), the camera failed to capture the corresponding picture due to the motion of the subject.

Figure 4B shows the motion amplitude changes of each limb node during the entire treatment process of the two patients. Patient 1’s motion amplitude at all key points is small except for the 7th treatment, which is consistent with the correlation between the severity of depressive symptoms and the decreased motion in previous studies. The motion amplitude of patient 2 remained stable during the entire course of psychotherapy, without obvious peaks and valleys. Patient 2’s motion amplitude is generally smaller, which is consistent with its low expression entropy and high frequency of sad expressions.

### Pearson correlation between BDD output and total scores of SDS, SAS, and HAMD of the two selected patients

**FIGURE 5:**
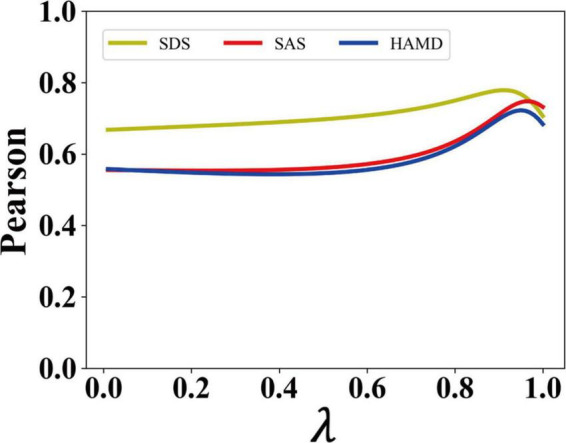
The Pearson similarity of the proposed model.

Figure 5 shows the Pearson similarity between BDD output from the model and clinically validated SDS, SAS, and HAMD scores, respectively. We adjust the proportion of expression entropy and action entropy in BDD by adjusting the parameter λ. In all tested cases, we found that when λ is 0.94, the mean Pearson similarity between BDD and each variate can reach more than 74%. This reveals the validity of our measurement model.

When λ is 0.94, we can calculate the BDD of patient 1 and patient 2 separately. As shown in Figure 2B, the BDD of patient 1 decreases continuously from 0.35 at baseline to 0.23 at the end of treatment, with a decrease of 34.3%. The overall trend is consistent with each clinically validated variate of patient 1. The BDD of patient 2 decreases from 0.47 at baseline to 0.35 at the end of treatment. After the 7th treatment, the BDD of patient 2 decreased by 25.5% due to the loss of suicidal thoughts. The overall trend is consistent with each clinically validated variate of patient 2. In addition, the BDD of patient 2 is lower than that of patient 1 because the depression severity of patient 2 is much more serious than that of patient 1. The trend in BDD of patient 1 and patient 2 is positively correlated with their clinically validated index of patient 1 and patient 2, with 74% Pearson similarity.

### Validation of BDD in all patients

**FIGURE 6:**
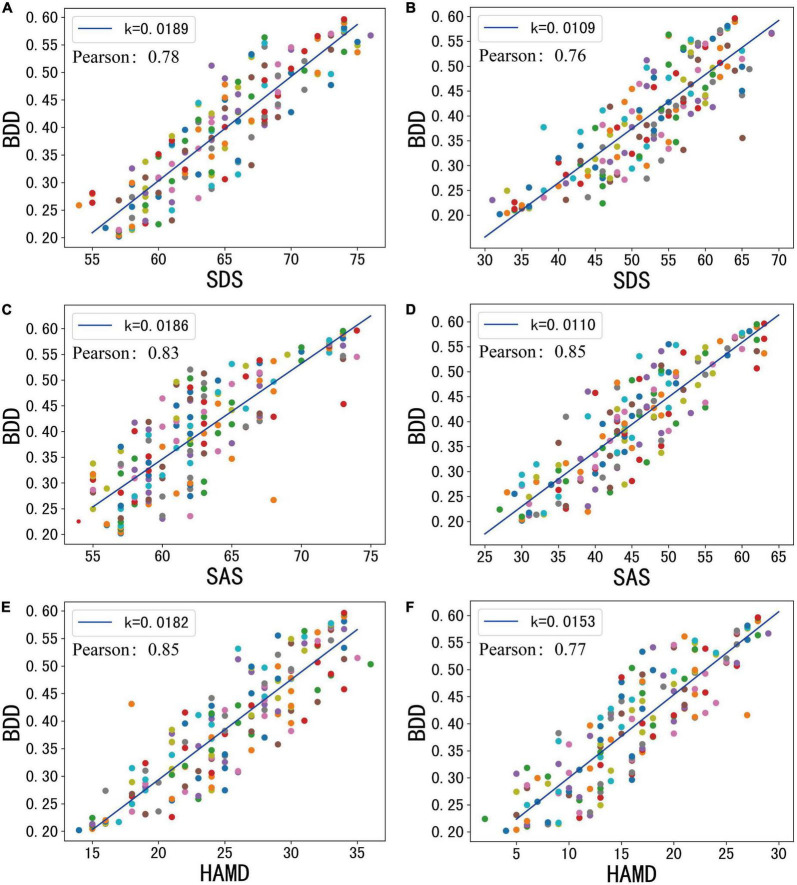
The total score of mental statistics scale and model test results before and after psychotherapy. **(A)** Baseline SDS. **(B)** End SDS. **(C)** Baseline SAS. **(D)** End SAS. **(E)** Baseline HAMD. **(F)** End HAMD.

In all 164 treatment cases, through the analysis of the patients’ condition with multiple treatment data, we found that their condition has been improved to a certain extent, but patients with various levels of improvement showed different performance data. The mean age of the patients was 25.2 with a standard deviation of 6.32, while their mean years of education was 14.8 with a standard deviation of 2.72. When λ is 0.94, the Pearson similarity between BDD and each variate can reach more than 74%, as shown in Figure 6.

At baseline, patients’ mean SDS, SAS, and HAMD were 64.6, 53.9, and 25.9, respectively. At the end of treatment, they were 53, 45.2, and 19.6, respectively. At baseline, the average BDD of the patients was 0.419. At the end of treatment, the average value of the patient’s BDD was 0.334. The BDD levels were all reduced by about 20%.

## Discussion

Previous AI detection models usually focus on a patient’s biological signals, such as blood pressure, pulse, EEG, and other physiological and biochemical information (41), or only consider unimodal appearance information such as facial expression (42, 43). In this manuscript, we proposed a multimodal deep convolution neural network to detect the severity of depression. Compared to an existing multimodal model (20, 31), our model has the following improvements: (i) we integrated facial expression information that is an important feature in evaluating the severity of depression; (ii) we constructed the BDD metrics to quantify the severity of depression and achieved a good performance; (iii) we found that the information extracted from different modes, when integrated in appropriate proportions, can significantly improve the accuracy of the evaluation, which has not been reported in previous studies.

We developed the BDD to measure the severity of depression symptoms in patients with MDD based on their facial expression and body movement, and analyzed the changes. Our study showed that the BDD is consistent with the SDS, SAS, and HAMD scores of patients with a 74% Pearson similarity. Through the progress of treatment, patients’ BDD decreased to various degrees after psychotherapy. This indicated that overall depression in the patients has been improved to a certain extent after treatment.

Although both the BDD and the patients’ symptoms showed that patients’ depressive symptoms were improved, the analysis showed that the probability of the patients’ sad expression was still significantly higher than other six expressions. This might be because it takes longer for the facial expression of depression to improve than the inner experience of depression. It might also be due to the complex etiology and pathogenesis of depressive disorder, such as genetics, personality, cognitive type, early life environment, social environment, and other factors, resulting in the “susceptibility” of some individuals to depression being higher than others (44).

Meanwhile, there is a link between the severity of depressive symptoms and facial expressions. The more severe the symptoms are, the more sad the expression is and the less the patient smiled (37). With the improvement of a depressed mood, depressed expression, and body motion range of patients can be improved accordingly (45). Facial expressions are the immediate manifestation of personal emotions. For patients with depression, a low mood and single facial expression are the primary symptoms. Anxiety, anger, pain, and other emotions caused by pressure are expressed through facial expressions (46), resulting in the facial expression as the major feature for the assessment of depression conditions.

We determined the proportion of facial expression and body movement in BDD in two patients, and found that when λ was 0.94, BDD effectively matched the patients’ scale trends, having a Pearson similarity exceeding 74% with the SDS, SAS, and HAMD scores. It is proposed that the BDD can effectively reflect the changes in a patient’s depressive condition, which shows a high degree of consistency with the trend of their psychological scale. At the same time, we verified that BDD could effectively fit the trend of SDS, SAS, and HAMD in all 164 patients. This demonstrated the validity of the fitting model in assessing depression conditions. In the process of information communication between people, facial expressions can convey 55% of the information while 38% of the information comes from pronunciation, intonation, and speech rhythm, and only 7% of the information comes from language content itself (47). This explains why our model has a large proportion of expression entropy.

In the future, research on the changes of facial expressions and motion amplitude of patients in the process of psychological treatment of depression based on deep learning algorithms can lay the foundation for the intelligent evaluation and monitoring of different kinds of treatment for patients dealing with depression.

## Conclusion

In conclusion, we presented a multi-modal deep learning method which combines expression and action features. The BDD proposed in this paper has demonstrated a beneficial function in effectively assessing the depression severity of MDD patients through the observation of their facial expressions and body movements.

This technique has significant applications in mental health treatments. It can monitor the symptoms of depression patients during psychotherapy and accurately assess the interaction between patients and therapists. At the same time, based on the patient’s psychological assessment, facial features, action features, and other data, we can develop a reliable deep learning evaluation model to train and process the patient’s multidimensional data, and evaluate the psychological changes of MDD patients accurately in real-time to guide the treatment. In addition, this study provides a new paradigm to research on depression and effectively solves the shortage of medical resources faced by patients with mental illness.

Although the model proposed in this paper has demonstrated its effectiveness in depression detection, there are still some limitations in the algorithm. Firstly, the accuracy of expression recognition and pose estimation needs to be further improved. For example, in future work, the 2D pose estimation algorithm can be replaced by 3D algorithms. Secondly, our multimodal model only includes two features: facial expressions and body movements. More features, such as voice tone, eye changes, and micro movements of patients can be considered in the future work. We hope this work will inspire others to build AI-based tools for understanding mental health disorders even beyond depression.

## Data availability statement

The raw data supporting the conclusions of this article will be made available by the authors, without undue reservation.

## Ethics statement

This study was approved by the Medical Ethics Committee of Second Xiangya Hospital of Central South University in China. All patients provided written informed consent prior to their participation in the study.

## Author contributions

DL: conceptualization, methodology, software, formal analysis, and writing, original draft. BL: data collection and writing – review and editing. TL: formal analysis and manuscript revision. GL: resources. GY: visualization and writing – review and editing. DQ, YQ, and OL: writing – review and editing. YL: validation. QY: investigation. SS: manuscript revision and editing. XL: visualization. XT: review and editing. JS and YC: supervision and project administration. HL: conceptualization, methodology, software, and manuscript revision. All authors contributed to the article and approved the submitted version.

## Footnotes

1
https://github.com/dlunion/DBFace

2
https://www.kaggle.com/datasets/deadskull7/fer2013

3
https://github.com/tryagainconcepts/tf-pose-estimation

## References

[1] Ferrari AJ Charlson FJ Norman RE Patten SB Freedman G Murray CJ Burden of depressive disorders by country, sex, age, and year: findings from the global burden of disease study 2010. *PLoS Med.* (2013) 10:e1001547. 10.1371/journal.pmed.1001547 24223526 PMC3818162

[2] Kessler RC Berglund P Demler O Jin R Koretz D Merikangas KR The epidemiology of major depressive disorder: results from the national comorbidity survey replication (NCS-R). *JAMA.* (2003) 289:3095–105. 10.1001/jama.289.23.3095 12813115

[3] Brundtland GH. Mental health: new understanding, new hope. *JAMA.* (2001) 286:2391. 10.1001/jama.286.19.2391 11712923

[4] Vos T Flaxman AD Naghavi M Lozano R Michaud C Ezzati M Years lived with disability (YLDs) for 1160 sequelae of 289 diseases and injuries 1990–2010: a systematic analysis for the global burden of disease study 2010. *Lancet.* (2012) 380:2163–96. 10.1016/S0140-6736(12)61729-2 23245607 PMC6350784

[5] Huang Y. Prevalence of mental disorders in China–author’s reply. *Lancet Psychiatry.* (2019) 6:468. 10.1016/S2215-0366(19)30177-4 31122476

[6] Cao Y Li W Shen J Malison RT Zhang Y Luo X. Health-related quality of life and symptom severity in Chinese patients with major depressive disorder. *Asia Pac Psychiatry.* (2013) 5:276–83. 10.1111/appy.12059 23857826

[7] Yuan Q Hong Z Wang X Shuai J Cao Y. Application of artificial intelligence in mental illness. *Int Psychiatry.* (2020) 47:4–7. 10.13479/j.cnki.jip.2020.01.002

[8] Patel M Andreescu C Price J Edelman K Reynolds C III Aizenstein H. Machine learning approaches for integrating clinical and imaging features in late-life depression classification and response prediction. *Int J Geriatr Psychiatry.* (2015) 30:1056–67. 10.1002/gps.4262 25689482 PMC4683603

[9] Redlich R Opel N Grotegerd D Dohm K Zaremba D Bürger C Prediction of individual response to electroconvulsive therapy via machine learning on structural magnetic resonance imaging data. *JAMA Psychiatry.* (2016) 73:557–64. 10.1001/jamapsychiatry.2016.0316 27145449

[10] Taguchi T Tachikawa H Nemoto K Suzuki M Nagano T Tachibana R Major depressive disorder discrimination using vocal acoustic features. *J Affect Disord.* (2018) 225:214–20. 10.1016/j.jad.2017.08.038 28841483

[11] Xu Z Zhang Q Li W Li M Yip PSF. Individualized prediction of depressive disorder in the elderly: a multitask deep learning approach. *Int J Med Inform.* (2019) 132:103973. 10.1016/j.ijmedinf.2019.103973 31569007

[12] Jiang H Dai Z Lu Q Yao Z. Magnetoencephalography resting-state spectral fingerprints distinguish bipolar depression and unipolar depression. *Bipolar Disord.* (2020) 22:612–20. 10.1111/bdi.12871 31729112

[13] Nemesure MD Heinz MV Huang R Jacobson NC. Predictive modeling of depression and anxiety using electronic health records and a novel machine learning approach with artificial intelligence. *Sci Rep.* (2021) 11:1–9. 10.1038/s41598-021-81368-4 33479383 PMC7820000

[14] Vincent P Mahendran N Nebhen J Deepa N Srinivasan K Hu Y-C. Performance assessment of certain machine learning models for predicting the major depressive disorder among it professionals during pandemic times. *Comput Intell Neurosci.* (2021) 2021:9950332. 10.1155/2021/9950332 33995524 PMC8096561

[15] Berenbaum H Oltmanns TF. Emotional experience and expression in schizophrenia and depression. *J Abnorm Psychol.* (1992) 101:37. 10.1037/0021-843X.101.1.37 1537971 PMC4370316

[16] Sloan DM Strauss ME Wisner KL. Diminished response to pleasant stimuli by depressed women. *J Abnorm Psychol.* (2001) 110:488. 10.1037/0021-843X.110.3.488 11502092

[17] Gao M Yang W Li C Chang Y Liu Y He Q Deep representation features from DreamDIAXMBD improve the analysis of data-independent acquisition proteomics. *Commun Biol.* (2021) 4:1–10. 10.1038/s42003-021-02726-6 34650228 PMC8517002

[18] Horstmann G. What do facial expressions convey: feeling states, behavioral intentions, or actions requests? *Emotion.* (2003) 3:150. 10.1037/1528-3542.3.2.150 12899416

[19] Liu B Shuai J Cao Y. Application of facial expression recognition technology in diagnosis and treatment of psychiatry. *Chin Behav Med Brain Sci.* (2021) 30:955–60. 10.3760/cma.j.cn371468-20201227-00084 30704229

[20] Dibeklioğlu H Hammal Z Cohn JF. Dynamic multimodal measurement of depression severity using deep autoencoding. *IEEE J Biomed Health Inform.* (2017) 22:525–36. 10.1109/JBHI.2017.2676878 28278485 PMC5581737

[21] Yang L Jiang D Han W Sahli H. DCNN and DNN based multi-modal depression recognition. *Proceedings of the 2017 Seventh International Conference on Affective Computing and Intelligent Interaction (ACII).* San Antonio, TX: IEEE Press (2017). p. 484–9. 10.1109/ACII.2017.8273643

[22] Gillespie S Laures-Gore J Moore E Farina M Russell S Haaland B. Identification of affective state change in adults with aphasia using speech acoustics. *J Peech Lang Hear Res.* (2018) 61:2906–16. 10.1044/2018_jslhr-s-17-0057 PMC6440307 30481797

[23] Jiang H Hu B Liu Z Wang G Zhang L Li X Detecting depression using an ensemble logistic regression model based on multiple speech features. *Comput Math Methods Med.* (2018) 2018:6508319. 10.1155/2018/6508319 30344616 PMC6174772

[24] Li X Zhong C-Q Wu R Xu X Yang Z-H Cai S RIP1-dependent linear and nonlinear recruitments of caspase-8 and RIP3 respectively to necrosome specify distinct cell death outcomes. *Protein Cell.* (2021) 12:858–76. 10.1007/s13238-020-00810-x 33389663 PMC8563874

[25] Lyu Z Wang Z Luo F Shuai J Huang Y. Protein secondary structure prediction with a reductive deep learning method. *Front Bioeng Biotechnol.* (2021) 9:687426. 10.3389/fbioe.2021.687426 34211967 PMC8240957

[26] Qian X Qiu Y He Q Lu Y Lin H Xu F A review of methods for sleep arousal detection using polysomnographic signals. *Brain Sci.* (2021) 11:1274. 10.3390/brainsci11101274 34679339 PMC8533904

[27] Qiu Y Liu D Yang G Qi D Lu Y He Q Cuffless blood pressure estimation based on composite neural network and graphics information. *Biomed Signal Process Control.* (2021) 70:103001. 10.1016/j.bspc.2021.103001

[28] Singh J Goyal G. Decoding depressive disorder using computer vision. *Multimed Tools Appl.* (2021) 80:8189–212. 10.1007/s11042-020-10128-9

[29] Wang S Shen Z Shen Z Dong Y Li Y Cao Y Machine-learning micropattern manufacturing. *Nano Today.* (2021) 38:101152. 10.1016/j.nantod.2021.101152

[30] Yuan Q Wang X Shuai J Lin H Cao Y. The application of artificial intelligence in depressive disorder. *Chin J Psychiatry.* (2020) 28:82–6. 10.16128/j.cnki.1005-3611.2020.01.019

[31] Othmani A Zeghina AO. A multimodal computer-aided diagnostic system for depression relapse prediction using audiovisual cues: a proof of concept. *Health Care Anal.* (2022) 2:100090. 10.1016/j.health.2022.100090

[32] Jeon J Park J-C Jo Y Nam C Bae K-H Hwang Y A real-time facial expression recognizer using deep neural network. *Proceedings of the 10th International Conference on Ubiquitous Information Management and Communication.* New York, NY: ACM (2016). p. 1–4. 10.1145/2857546.2857642

[33] Carreira J Agrawal P Fragkiadaki K Malik J. Human pose estimation with iterative error feedback. *Proceedings of the IEEE Conference on Computer Vision and Pattern Recognition.* Las Vegas, NV: (2016). p. 4733–42. 10.1109/CVPR.2016.512

[34] Hamm J Kohler CG Gur RC Verma R. Automated facial action coding system for dynamic analysis of facial expressions in neuropsychiatric disorders. *J Neurosci Methods.* (2011) 200:237–56. 10.1016/j.jneumeth.2011.06.023 21741407 PMC3402717

[35] Hamm J Pinkham A Gur RC Verma R Kohler CG. Dimensional information-theoretic measurement of facial emotion expressions in schizophrenia. *Schizophr Res Treatment.* (2014) 2014:1–10. 10.1155/2014/243907 24724025 PMC3956414

[36] Goodfellow IJ Erhan D Carrier PL Courville A Mirza M Hamner B Challenges in representation learning: a report on three machine learning contests. *Proceedings of the International Conference on Neural Information Processing.* Berlin: Springer (2013). p. 117–24. 10.1007/978-3-642-42051-1_16

[37] Girard JM Cohn JF Mahoor MH Mavadati S Rosenwald DP. Social risk and depression: evidence from manual and automatic facial expression analysis. *Proceedings of the 2013 10th IEEE International Conference and Workshops on Automatic Face and Gesture Recognition (FG).* Shanghai: IEEE (2013). p. 1–8. 10.1109/fg.2013.6553748 PMC3935843 24598859

[38] Felzenszwalb PF Huttenlocher DP. Pictorial structures for object recognition. *Int J Comput Vis.* (2005) 61:55–79. 10.1023/B:VISI.0000042934.15159.49

[39] Yang W Wang Y Mori G. Recognizing human actions from still images with latent poses. *Proceedings of the 2010 IEEE Computer Society Conference on Computer Vision and Pattern Recognition (IEEE).* San Francisco, CA: (2010). p. 2030–7. 10.1109/CVPR.2010.5539879

[40] Horigome T Sumali B Kitazawa M Yoshimura M Liang K Tazawa Y Evaluating the severity of depressive symptoms using upper body motion captured by RGB-depth sensors and machine learning in a clinical interview setting: a preliminary study. *Compr Psychiatry.* (2020) 98:152169. 10.1016/j.comppsych.2020.152169 32145559

[41] Mumtaz W Ali SSA Yasin MAM Malik AS. A machine learning framework involving EEG-based functional connectivity to diagnose major depressive disorder (MDD). *Med Biol Eng Comput.* (2018) 56:233–46. 10.1007/s11517-017-1685-z 28702811

[42] Guo W Yang H Liu Z Xu Y Hu B. Deep neural networks for depression recognition based on 2d and 3d facial expressions under emotional stimulus tasks. *Front Neurosci.* (2021) 15:609760. 10.3389/fnins.2021.609760 33967675 PMC8102822

[43] Lee Y-S Park W-H. Diagnosis of depressive disorder model on facial expression based on fast R-CNN. *Diagnostics.* (2022) 12:317. 10.3390/diagnostics12020317 35204407 PMC8871079

[44] Harrison P Cowen P Burns T Fazel M. *Shorter Oxford Textbook of Psychiatry.* Oxford: Oxford University Press (2017).

[45] Szabadi E Bradshaw C Besson J. Elongation of pause-time in speech: a simple, objective measure of motor retardation in depression. *Br J Psychiatry.* (1976) 129:592–7. 10.1192/bjp.129.6.592 1000144

[46] Ding Y Dai J. Advance in stress for depressive disorder. *Adv Exp Med Biol.* (2019) 1180:147–78. 10.1007/978-981-32-9271-0_8 31784962

[47] Mehrabian A Russell JA. *An Approach to Environmental Psychology.* Cambridge, MA: The MIT Press (1974).

